# Solitary Fibrous Tumor in the Orbit: A Rare Occurrence

**DOI:** 10.7759/cureus.88793

**Published:** 2025-07-26

**Authors:** Jai Sethi, Arpit M Chhabra, Athena M Loiacono, Thomas R Eanelli

**Affiliations:** 1 Radiation Oncology, Northeastern University, Boston, USA; 2 Radiation Oncology, New York Proton Center, New York, USA; 3 Basic Biomedical Sciences, Touro College of Osteopathic Medicine, Middletown, USA; 4 Radiation Oncology, Garnet Health, Middletown, USA

**Keywords:** orbital tumor, proton beam therapy, radiation oncology education, solitary fibrous tumor (sft), targeted radiation therapy

## Abstract

Solitary fibrous tumors (SFTs) of the orbit are exceptionally rare neoplasms. We present the case of a 77-year-old female who first presented in 2021 with a spindle cell neoplasm in the superior right orbit. Following surgical excision and a negative PET scan, she opted for surveillance without adjuvant radiation therapy. Three years later, MRI revealed a recurrence of the orbital SFT. The patient underwent a second surgical resection and adjuvant proton radiation therapy to reduce the risk of further recurrence. This case draws attention to the importance of long-term follow-up in orbital SFTs and highlights the potential role of targeted proton therapy for managing recurrent cases.

## Introduction

Orbital tumors account for an extremely small number of cases in the field of oncology. Out of all tumors of the orbit, 68% are benign and 32% are malignant [1]. Solitary fibrous tumors (SFTs) are rare mesenchymal tumors that can arise in various anatomical locations, with a majority occurring in the pleura [2]. Less commonly, they have also been identified in areas like the mediastinum, liver, and retroperitoneum, among others; when diagnosed, SFTs are normally benign and rarely metastasize [3,4]. SFTs are known to affect both sexes equally as well as all ages of patients, with a slight predominance to middle-aged adults [5].

Orbital SFTs are especially uncommon and present unique diagnostic and management challenges due to their location and potential for recurrence. Classified by the World Health Organization as tumors of “intermediate malignancy,” SFTs typically grow slowly and display benign behavior but can sometimes recur or metastasize, particularly in rare sites like the orbit [6]. In the latest WHO classification (5th edition 2020), SFTs are subdivided into benign SFT (intermediate category (locally aggressive)), SFT NOS (intermediate category (rarely metastasizing)), and malignant SFT. Likewise, in cases of SFTs that have metastasized to the orbit, most behave in a benign manner; however, some display behavior of a malignant tumor, such as with recurrence and metastasis [7].

In the orbit, SFTs can mimic other orbital masses clinically and radiologically, making diagnosis reliant on histopathological and immunohistochemical analysis [8]. These tumors are generally positive for CD34, STAT6, and other markers that help differentiate them from other spindle cell neoplasms [9]. Only a limited number of orbital SFT cases have been documented in the literature, with about 150 cases having been reported since 2024 and even fewer cases detailing recurrence after initial surgical management [10].

We present a rare case of recurrent SFT in the superior right orbit of a 77-year-old female. Initially managed with surgical resection and surveillance, her recurrent case now presents an opportunity to explore the role of proton radiation therapy in preventing further recurrence. This report discusses the diagnostic and therapeutic considerations for recurrent orbital SFTs and the implications of advanced targeted therapy in improving patient outcomes.

## Case presentation

In February 2021, a 73-year-old female patient with a medical history of endometrial cancer, bursitis, and meningioma presented to her provider, reporting a sensation of an object lodged in her right eye. Over the following months, she developed a right upper eyelid lesion, initially believed to be a stye, which did not respond to conventional stye treatments.

Initial presentation and diagnosis

**Figure 1:**
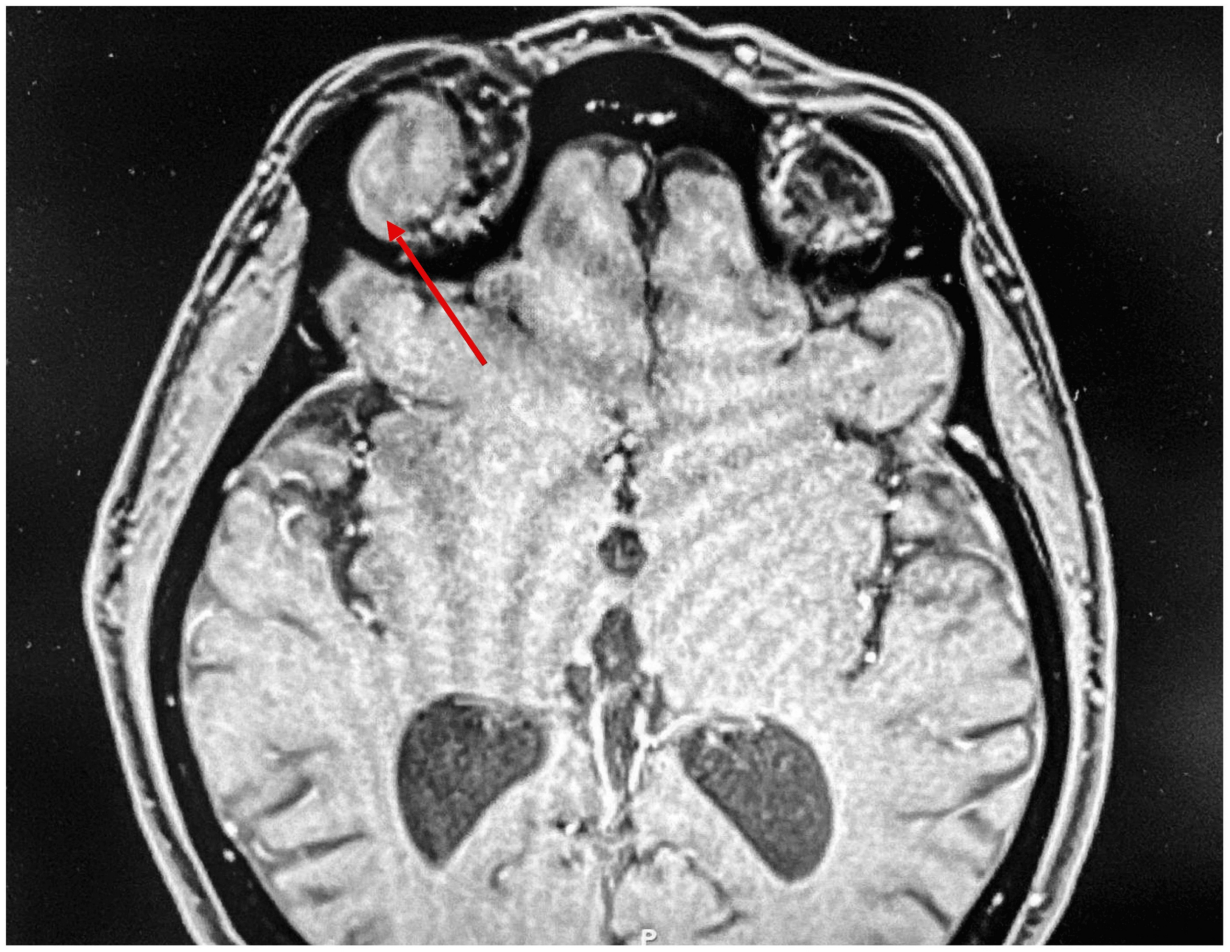
Diagnostic image showing a solitary fibrous tumor in the superior right orbit of the patient.

By June 2021, the patient experienced double vision, prompting further evaluation. A repeat CT in May 2021 revealed a 1.0 x 1.4 x 2.8 cm amorphous hyperdense lesion in the superior portion of the right orbit (Figure 1). On September 8, 2021, the patient underwent a surgical excision of the tumor, and pathology confirmed the mass as a spindle cell neoplasm, a rare orbital tumor subtype.

Postoperative management and surveillance

In October 2021, the patient underwent a full-body scan, which showed no evidence of metastatic disease in the chest, abdomen, or pelvis. A follow-up PET scan on November 22, 2021, was also negative. Although postoperative radiation therapy was recommended, the patient chose active surveillance over immediate radiation treatment.

Recurrence and second surgical intervention

In March 2024, the patient began experiencing recurrent symptoms, leading to an MRI that revealed a 2.3 x 1.9 x 0.8 cm homogeneously enhancing extraconal mass in the right orbit. This image finding indicated a recurrence of the spindle cell neoplasm. Consequently, on May 1, 2024, the patient underwent a second surgical excision. Immunohistochemistry confirmed the recurrent tumor as a spindle cell neoplasm, with immunopositivity for CD31 (80-90%), CD34 (>90%), ERG (5-10%), and STAT6 (>90%).

Radiation therapy consultation and planning

**Figure 2:**
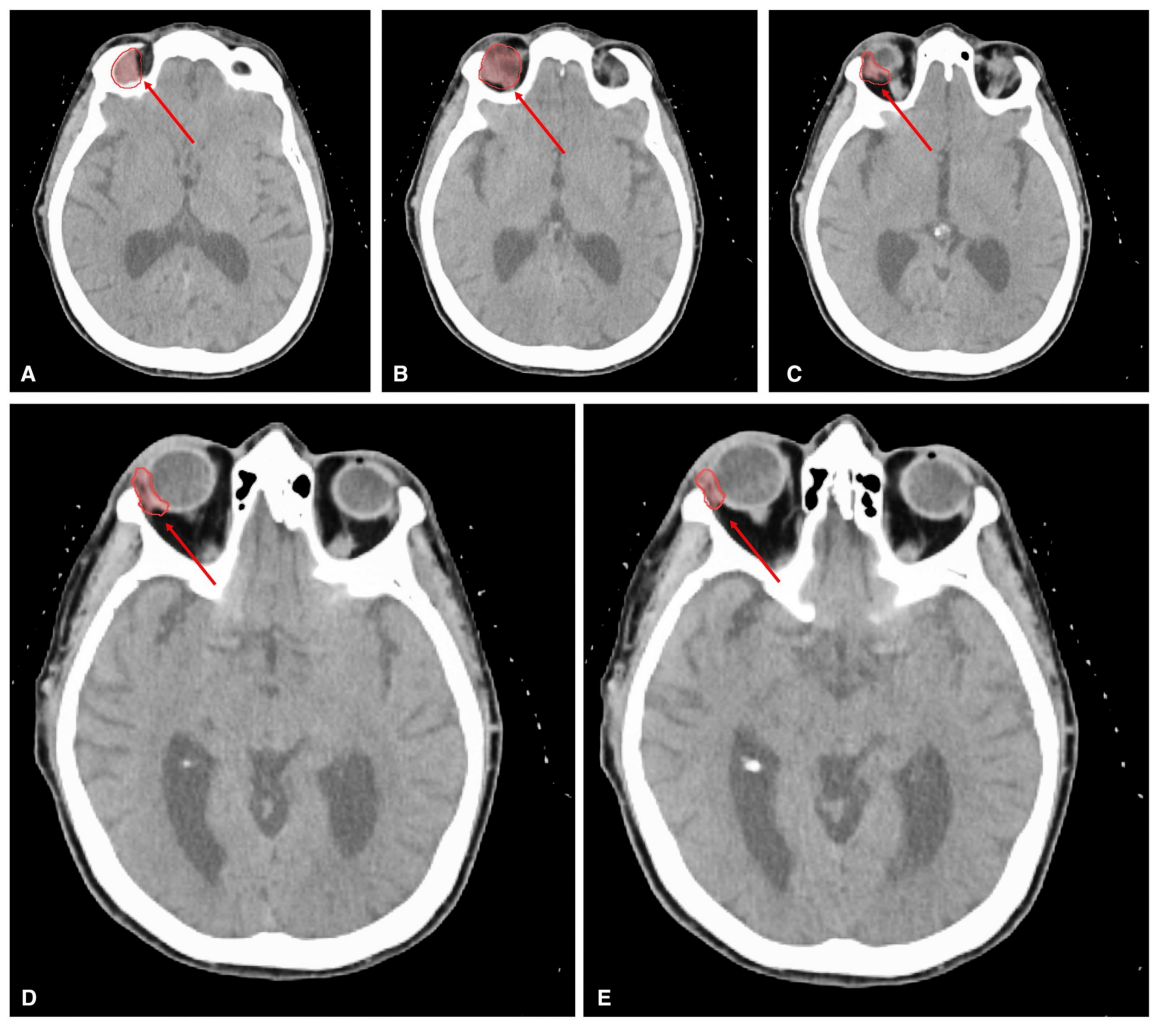
Images of proton radiation therapy plan. Red contours indicate the clinical target volume and represent the tumor target to which 60 Gy of radiation has been prescribed. A, B, C, D, and E display images of the tumor at different CT slices.

**Figure 3:**
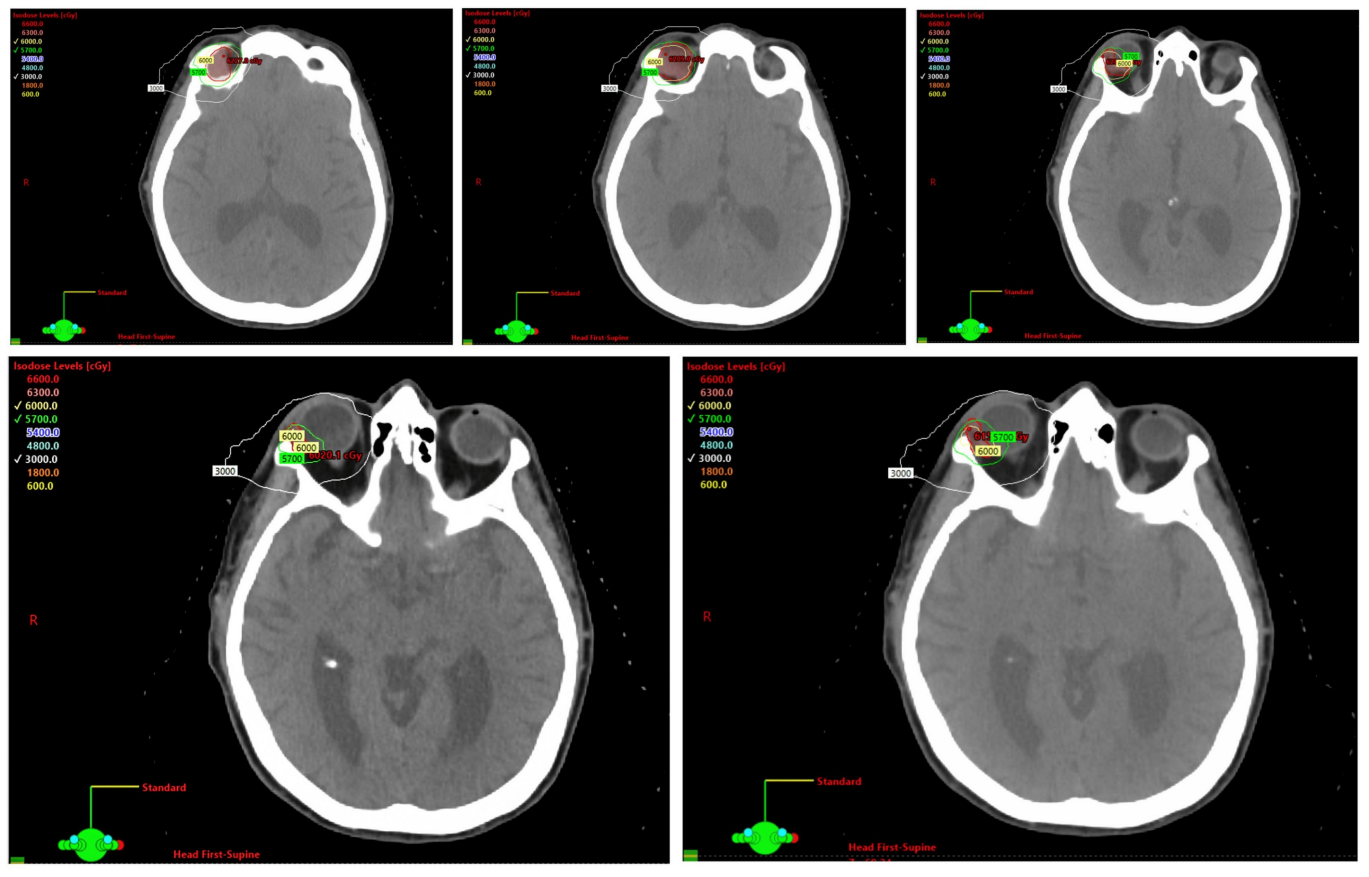
Isodose planning images. Images display the isodose lines, which correspond to 60 Gy (100% of prescription dose), 57 Gy (95% isodose line), and 30 Gy (50% isodose line) to show the stopping power of protons and the ability to spare surrounding normal tissues (brain, contralateral eye, nerves).

Following the recurrence, the patient was referred for consultation at the New York Proton Center, where she discussed targeted radiation therapy options with her care team. Given the tumor’s sensitive orbital location, proton therapy was deemed the optimal treatment to minimize damage to surrounding tissues, including the brain, contralateral eye, and optic nerves. Treatment planning images (Figure 2) illustrate the targeted clinical volume, prescribed to a dose of 60 Gy proton dose. The isodose lines (Figure 3) highlight the precision of proton therapy, demonstrating its ability to effectively target the tumor while sparing critical surrounding structures.

Upon meeting with the team at the New York Proton Center, the patient discussed the best course of action with her providers and made the decision to undergo proton radiation therapy. The image in Figure 2 shows the plan for proton radiation therapy, with the red circles indicating the tumor target. The plan is for the tumor target to be treated with a prescribed dose of 60 Gy of radiation.

## Discussion

This case of a 77-year-old female with an SFT of the superior right orbit and a history of endometrial cancer and meningioma presents various diagnostic and therapeutic challenges. SFTs in the right orbit, originally believed to be uncommon, are now being shown to be increasingly prevalent. To our knowledge, the first case of SFT in the orbit documented in the literature was reported in 1994 by Westra et al. [11]. Since this publication, there has been a steady increase in cases reported in the literature over the past 30 years, with the total number of cases believed to be above 150.

Previous studies have shown that complete surgical resection followed by long-term neuroimaging and follow-up is necessary for adequate surveillance [11]. The patient in this case opted for this pathway of management following her first surgical resection, leading to the discovery and subsequent surgical removal of the recurrent SFT.

It is important to emphasize how unusual the location of this recurrent SFT is. For the most part, SFTs have been found in areas including the pelvis, groin, and retroperitoneum [12]. Orbital SFTs are not common, and there are only a handful of cases that have been reported. These tumors are unique due to their mesenchymal origin and the aforementioned WHO classification. These tumors, especially in the orbit, are rarely recurrent. In some cases, even after initial surgical resection, recurrences can occur due to a variety of factors. A previous study discovered that certain factors, such as high mitotic index, Ki67 index, and the presence of necrosis, led to an increase in the risk of recurrence following a surgically resected SFT [13].

As previously stated, immunohistochemistry findings confirmed the recurrent tumor as a spindle cell neoplasm, with immunopositivity for CD31 (80-90%), CD34 (>90%), ERG (5-10%), and STAT6 (>90%). These findings are important for spindle cell tumors. The immunopositivity markers help distinguish SFTs from other orbital tumors. For example, in the case of STAT6, which was immunopositive in the recurrent tumor, nuclear expression can be found in almost all cases related to SFTs, but is limited in other examples of soft tissue neoplasms. As a result, STAT6 is incredibly sensitive and is a nearly perfect marker specifically for SFTs [14].

When comparing this case to the handful of previously reported cases of SFTs in the orbit, there are some important similarities and differences of note. Most cases of orbital SFTs reported have been in younger age groups (median 42 years old), while the patient in this case report is 77 years of age. These tumors have been shown to affect both sexes equally. Like the patient in this case, an expanding/swelling of the eyelid was the most common symptom. Unlike this case, blurred vision is typically not seen [15].

As previously mentioned, the patient has opted for proton therapy rather than traditional radiation therapy. Proton therapy offers several advantages, especially when dealing with orbital tumors. The orbit is an incredibly delicate area in the human body, and precision is crucial in order to minimize damage to the surrounding tissues (i.e., brain, contralateral eye, nerves, etc.). Proton radiation therapy has been used for the complete sparing of tissue beyond the target volume [16]. Another important topic of note is the Bragg peak effect, which reduces radiation exposure to sensitive surrounding tissues. The Bragg peak is a pronounced peak on the Bragg curve that, for protons, alpha rays, and ion rays, occurs immediately before the particles come to rest [17]. The Bragg peak can be spread out in depth using specialized filters to achieve the desired dose deposition pattern. As a result, it is then possible to design a three-dimensional dose deposition that is confined precisely to the tumor volume, with an extremely sharp dose fall-off to the normal tissue distal to the tumor [18].

Challenges in management also occur when dealing with orbital SFTs. As in this case, there is always the potential for recurrence despite a complete surgical resection. There are also challenges in decision-making regarding radiation therapy following initial observation. When dealing with rare tumor sites such as the orbit, treatment options are limited, making it difficult to determine the best course forward for many patients. Given the incredibly rare nature of orbital SFTs, it is important for case reports to be published in order to expand upon the established knowledge base on the topic. Future studies can focus on long-term outcomes with orbital SFTs and can delve into advanced radiotherapy techniques in order to prevent recurrence.

## Conclusions

This case highlights the clinical challenges of managing recurrent SFTs in sensitive and rare anatomical sites such as the orbit. Our patient, a 77-year-old female, experienced recurrence of an orbital SFT three years post-initial surgical resection, illustrating the potential for recurrence despite a histologically intermediate malignancy classification. Her decision to undergo proton radiation therapy draws attention to the importance of advanced, targeted treatment options to preserve function and minimize damage to critical surrounding structures, particularly in complex anatomical areas.

The rarity of orbital SFTs, combined with the unpredictable behavior of these tumors, emphasizes the need for continued documentation of similar cases to better understand recurrence patterns and refine treatment strategies. This case also demonstrates the evolving role of proton therapy in oncology, particularly for tumors in close proximity to critical structures, where precision is paramount.

Future studies should focus on long-term outcomes for patients with orbital SFTs treated with proton therapy, as well as on the development of comprehensive guidelines for post-surgical management of recurrent cases. Documenting and disseminating such cases can contribute to building a more substantial knowledge base for these rare tumors, ultimately improving patient care and outcomes.
